# Clinical and dosimetric predictors of radiation-induced rhinosinusitis following VMAT for nasopharyngeal carcinoma: A retrospective study

**DOI:** 10.1016/j.heliyon.2023.e23554

**Published:** 2023-12-12

**Authors:** Xiaomin Bao, Yan Wang, Bin Li, Liang Peng, Bin Ouyang, Chew Lip Ng, Yongshi Zhuo, Qiumin Wang, Chunwei Li, Jian Li

**Affiliations:** a Department of Otorhinolaryngology, The First Affiliated Hospital of Sun Yat-sen University, Guangdong, Guangzhou, China; b Guangxi Hospital Division of The First Affiliated Hospital, Sun Yat-sen University, Guangxi, Nanning, China; c Department of Radiation Oncology, The First Affiliated Hospital, Sun Yat‐sen University, Guangdong, Guangzhou, China; d Clinical Trials Unit, The First Affiliated Hospital, Sun Yat‐sen University, Guangdong, Guangzhou, China; e Department of Otolaryngology-Head and Neck Surgery, Ng Teng Fong General Hospital, National University Health System, Singapore

**Keywords:** Radiation-induced rhinosinusitis, Volumetric-modulated arc therapy, Dosimetric predictors, Nasopharyngeal carcinoma

## Abstract

**Background:**

We aimed to investigate the clinical and dosimetric factors associated with radiation-induced rhinosinusitis, and further elucidate the optimal dose-volume constraints for nasopharyngeal cancer patients who underwent volumetric-modulated arc therapy (VMAT).

**Methods:**

A retrospective review of 196 nasopharyngeal carcinoma (NPC) patients who underwent definitive VMAT between August 2018 and May 2021 was conducted. Both clinical and dose-volume histogram (DVH) data of NPC patients without rhinosinusitis at baseline were selected for analysis.

**Results:**

The cumulative incidence of post-RT rhinosinusitis at the 3-, 6-, 9-, and 12-months, and >1 year were 29.6 %, 41.3 %, 42.9 %, and 45.4 %, and 47.4 %, respectively. Nasal irrigation was negatively associated with post-RT rhinosinusitis (p < 0.001). Higher cumulative incidences of maxillary and ethmoid sinusitis were associated with V70 > 1.16 % and >1.00 %, respectively (p = 0.027 and p = 0.002). Sphenoid sinusitis was more frequent when Dmax(maxillary sinus) exceeded 69.2Gy (p = 0.005).

**Conclusions:**

Regular nasal irrigation may reduce the development of rhinosinusitis. Dose-volume constraints of V70 and Dmax to the maxillary sinus are suggested for VMAT planning. Patients exceeding these thresholds should be closely monitored and potentially offered preventative interventions within 3–6 months post-RT.

## 1 Introduction

Nasopharyngeal carcinoma (NPC) is prevalent in Southern China [1]. Radiotherapy (RT) represents the standard treatment for NPC, owing to the anatomical location of the nasopharynx and the high radiosensitivity of cancer cells. Advancements in RT, alongside the development of effective systemic chemotherapy regimens, have allowed for excellent locoregional control in the past two decades [2,3]. The development of three-dimensional (3D) conformal RT, and intensity-modulated radiation therapy (IMRT) have resulted in reduced radiation-related toxicity and improved treatment outcomes [4,5]. However, treatment-related complications such as oral mucositis, dry mouth, trismus, and rhinosinusitis remain a concern. There is high incidence of radiation-induced sinus mucosa disease (SMD) among NPC patients following RT [6], with that of rhinosinusitis reported up to 73.5 % following IMRT [7]. Studies have further shown higher incidence of post-irradiation cytological and olfactory changes such as squamous metaplasia, subepithelial oedema, ciliary dysmotility, and nasopharyngeal secretion among NPC patients compared to those with common chronic rhinosinusitis without nasal polyps [8,9]. Symptoms of rhinosinusitis including purulent discharge, nasal congestion, nasal obstruction, hyposmia, and headache may not only affect the quality of life of NPC patients, but also pose an economic burden to both patients and the healthcare system [10].

Advanced tumor stage, smoking habit, nutritional status, and nasal cavity invasion have been identified as risk factors for rhinosinusitis in NPC patients [7,11]. In terms of radiation dose thresholds, Yin et al. have reported ≤37 Gy as an optimal mean dose for preservation of nasal cavity function [12]. While Su et al. reported that exposure to dosage ≥72 Gy increased the incidence of rhinosinusitis [7]. While prior studies have established dose thresholds, they primarily focused on nasal mean doses or nasopharyngeal tumor irradiation doses, without a comprehensive analysis of sinus dose distribution. With VMAT's advancement that enhances target dose conformity while conserving adjacent critical structures over IMRT [13], its impact on post-RT rhinosinusitis, especially considering the maxillary sinus where the highest incidence of sinusitis occurs, has yet to be thoroughly investigated.

Our study goals were to elucidate both the clinical characteristics and dosimetric risk factors, to identify the dose-volume thresholds for radiation-induced rhinosinusitis following VMAT for NPC, and to guide individualized preventative and appropriate treatment interventions in such patients.

## 2 Materials and methods

### 2.1 Study design, setting, participants and ethical considerations

A retrospective analysis of 196 NPC patients treated in The Institute of Radiation Oncology of our hospital, Guangzhou, Southern China, between August 2018 and May 2021 was conducted. Demographic parameters (sex and age), the relevant clinical data (NPC staging and treatment regimes), medical history (asthma, alcohol, allergies rhinitis, nasal irrigation and so on), and dosimetric parameters were recorded.

**Fig. 1:**
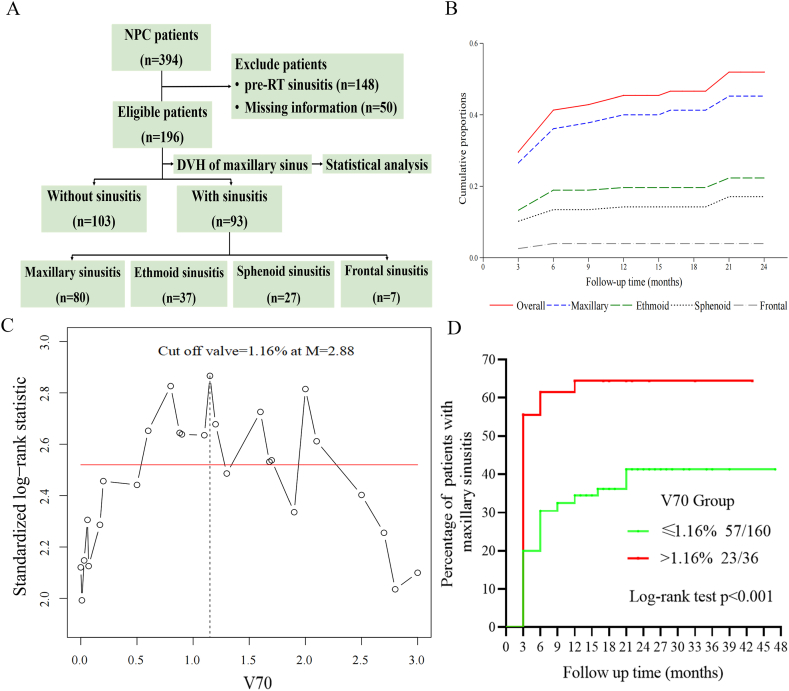
**(**A) Flow diagram of patient exclusion and inclusion. (B) Cumulative incidence of post-RT rhinosinusitis. (C) Absolute standardized log-rank statistics for the predictor V70 and significance bound based on the improved Bonferroni inequality. (D) Kaplan-Meier curves of two groups of NPC patients separated by the cutpoint 1.16 % V70.

The inclusion criteria were containing 3 points: (1) histologically diagnosed primary NPC; (2) presence of computed tomography (CT) or magnetic resonance imaging (MRI) scan documenting the axial and coronal positions of the nasopharynx and tumour, and effect after VMAT; and (3) >1-year follow-up without rhinosinusitis developed within 1 year of definitive VMAT. The exclusion criteria from the study were as follows: (1) history of sinus surgery; (2) presence of pre-RT rhinosinusitis. Originally, 394 NPC patients treated in The Department of Radiation Oncology of our hospital, but 148 (37.5 %) had pre-RT rhinosinusitis, and 50 (12.7 %) had incomplete data. As such, 196 were eventually included in our analysis (Fig. 1A).

All patients were subjected to standard diagnostic protocols. CT were acquired in 0.625 mm slice thickness, and MRI images was administered by using the Siemens Verio 3.0 T scanner (Siemens SOMATOM Force, Forchheim, Germany). NPC was staged based on the 8th edition of the Union for International Cancer Control/American Joint Committee on Cancer (UICC/AJCC) staging system. The retrospective observational trial was approved by IEC for Clinical Research and Animal Trials of our University (2022265).

### 2.2 Main outcomes measures

#### 2.2.1 Treatment

##### 2.2.1.1 Chemotherapy

Chemotherapy was administered in accordance with the treatment protocol of our institution. Most patients received concurrent chemotherapy (n = 171), while all patients with tumor stage I (n = 3), and a proportion of those with tumor stage Ⅱ (n = 22), were treated with radiotherapy alone (n = 25). Patients with stages Ⅲ-Ⅳ NPC were treated with either combined induction or concurrent chemotherapy.

##### 2.2.1.2 Radiotherapy

All patients received definitive VMAT. We performed delineation of the target volume according to the institutional treatment guidelines [14,15]. The total radiation doses to the planning target volume (PTV) of the primary gross tumor volume (GTVnx) were 68–76 Gy/30–31 fractions, 60–66 Gy/30–31 fractions for the PTV of the GTV of involved lymph node (GTVnd), 60–62 Gy/30–31 fractions to the PTV of high‐risk clinical target volumes (CTV1), and 54–56 Gy/30–31 fractions to the PTV of low‐risk clinical target volumes (CTV2).

#### 2.2.2 DVH parameters of the maxillary sinus

**Fig. 2:**
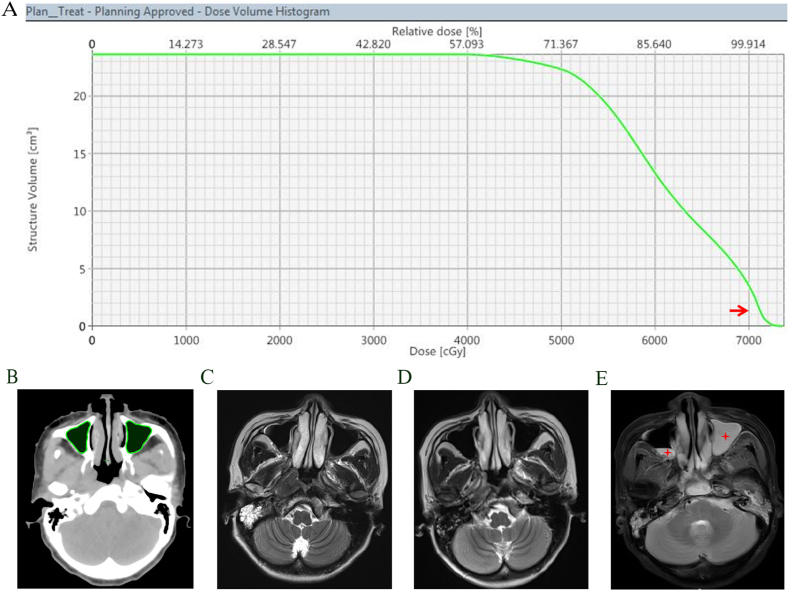
**(**A) Dosimetric parameters of the maxillary sinus (V70 = 1.16 %, red arrow). (B) Manual contours of the maxillary sinus. MRI images (C) pre-RT, (D) 3 months post-RT, and (E) 6 months post-RT (maxillary sinusitis, red star). (For interpretation of the references to colour in this figure legend, the reader is referred to the Web version of this article.)

Based on the Eclipse v13.6 planning system (Varian Medical Systems, USA) and Varian Trilogy accelerator, VMAT was planned for each patient on the CT simulation images prior to RT. Manual contouring of the maxillary sinus was done by a single physician via the treatment planning system. A multilayer gated recurrent unit-based recurrent neural network (GRU-RNN) inputs method was used to accurate Dose‐volume histograms (DVH) prediction. DVH of the maxillary sinus were derived from 3D dose distribution. DVH data, including mean (Dmean), minimum (Dmin), maximum (Dmax) and median (Dmed) radiation doses to the maxillary sinus, absolute maxillary sinus volume, the percentage of maxillary sinus volume receiving greater than the designated radiation dose (V20Gy, V30Gy, V40Gy, V50Gy, V60Gy, V70Gy) were collected for statistical analysis (Fig. 2A and B).

#### 2.2.3 Diagnostic criteria of rhinosinusitis

Evidence of rhinosinusitis was determined based on CT or MRI images [16]. The diagnostic criteria included (1) sinus mucosa thickening on enhanced scans (≥3 mm); (2) regions of hyperdensity in the sinus; (3) air-fluid level in the sinus cavity [17]. Rhinosinusitis was considered in the presence of inflammation at least one side of either the maxillary, ethmoid, sphenoid, or frontal sinus. Maxillary sinusitis was diagnosed in the presence of inflammation at least one side of either the maxillary sinus (Fig. 2C–E).

#### 2.2.4 Follow-up

The last follow-up date for our series ranged from the date of completion of VMAT to the date of last clinical visit or 30 May 2022, whichever came first. MRI evaluations were performed every 3–6 months in first 2 years, and every 6–12 months thereafter.

### 2.3 Statistical analyses

All statistical analyses were performed using the SPSS 25.0 software package (Chicago, IL, USA) and R software version 0.94.102. Continuous variables were compared using the Mann-Whitney *U* test, while categorical variables were compared using the Chi-square (χ2) or Fisher's exact test. Age was categorized according to the median age (>48 years vs. ≤48 years). The logistic regression model was used to analyze the correlations of post-RT rhinosinusitis with age, sex, TNM stage, treatment regime, hypertension, diabetes mellitus, nasal irrigation, and dosimetric parameters. Multivariate analyses were performed on variables with p < 0.05 from the univariate analysis. Maximally selected log-rank statistics pinpointed independent predictors, with receiver operating characteristic (ROC) curves determining dose-volume thresholds. The Kaplan-Meier method was used to analyze the cumulative incidence of post-RT rhinosinusitis. A p < 0.05 was considered statistically significant.

## 3 Results

### 3.1 Clinical characteristics and incidence of post-RT rhinosinusitis

Among the 196 patients included in the study, 136 were male and 60 were female. The median age was 48 years (range, 15–78 years). The median follow-up period was 12 months (range, 3–47 months). Post-RT rhinosinusitis was observed in 93 (47.7 %) patients. The cumulative incidence of rhinosinusitis at 3, 6, 9, and 12 months, and >1 year post-RT were 29.6 %, 41.3 %, 42.9 %, 45.4 % and 47.4 %, respectively (Fig. 1B). Maxillary sinusitis (40.8 %) was most commonly observed, followed by ethmoid sinusitis (18.9 %) and sphenoid sinusitis (13.8 %). The clinical characteristics of the patients are summarized in Table 1, while the DVH parameters are shown in Table 2.

**Table 1:** Clinical characteristics and the χ2 test of patients with NPC.

Variable	Patients No. (%)		p	Patients No. (%)		p
	With rhinosinusitis (n = 93)	Without rhinosinusitis (n = 103)		With maxillary sinusitis (n = 80)	Without maxillary sinusitis (n = 116)	
Age (y)						
≤48	44 (22.45)	46 (23.47)	0.710	36 (18.37)	54 (27.55)	0.830
＞48	49 (25.00)	57 (29.08)		44 (22.45)	62 (31.63)	
Sex						
Female	28 (14.29)	32 (16.33)	0.432	22 (11.22)	78 (39.80)	0.434
Male	65 (33.16)	71 (36.22)		58 (29.59)	38 (19.39)	
Tumor stage^a^						
T1	5 (2.55)	12 (6.12)	0.145	4 (2.04)	13 (6.63)	0.218
T2	24 (12.24)	32 (16.33)		21 (10.71)	35 (17.86)	
T3	50 (25.51)	40 (20.41)		43 (21.94)	47 (23.98)	
T4	14 (7.14)	19 (9.69)		12 (6.12)	21 (10.71)	
Node stage^a^						
N0	8 (4.08)	9 (4.59)	0.952	6 (3.06)	11 (5.61)	0.843
N1	45 (22.96)	53 (27.04)		38 (19.39)	60 (30.61)	
N2	24 (12.24)	23 (11.73)		21 (10.71)	26 (13.27)	
N3	16 (8.16)	18 (9.18)		15 (7.65)	19 (9.69)	
Overall stage^a^						
I-Ⅱ	14 (7.14)	24 (12.24)	0.145	11 (5.61)	27 (13.78)	0.097
Ⅲ-Ⅳ	79 (40.31)	79 (40.31)		69 (35.20)	89 (45.41)	
Metastasis stage^a^						
M0	92 (46.94)	103 (52.55)	0.474	79 (40.31)	116 (59.18)	0.408
M1	1 (0.51)	0 (0)		1 (0.51)	0 (0)	
WHO pathologic						
Undifferentiated	93 (47.45)	103 (52.55)	–	80 (40.82)	116 (59.18)	–
Treatment regime						
RT alone	9 (4.59)	16 (8.16)	0.220	8 (4.08)	17 (8.67)	0.337
Chemotherapy	84 (42.86)	87 (44.39)		72 (36.73)	99 (50.51)	
Alcohol						
No	82 (41.84)	92 (46.94)	0.799	69 (35.20)	105 (53.57)	0.352
Yes	11 (5.61)	11 (5.61)		11 (5.61)	11 (5.61)	
Smoker						
No	64 (32.65)	66 (33.67)	0.483	53 (27.04)	77 (39.29)	0.985
Yes	29 (14.80)	37 (18.88)		27 (13.78)	39 (19.90)	
Hypertension						
No	88 (44.90)	98 (50)	0.868	75 (38.27)	111 (56.63)	0.544
Yes	5 (2.55)	5 (2.55)		5 (2.55)	5 (2.55)	
Diabetes mellitus						
No	91 (46.43)	101 (51.53)	0.918	78 (39.80)	114 (58.16)	0.706
Yes	2 (1.02)	2 (1.02)		2 (1.02)	2 (1.02)	
Asthma						
No	93 (47.45)	103 (52.55)	–	80 (40.82)	116 (58.18)	–
Yes	0 (0)	0 (0)		0 (0)	0 (0)	
Allergies rhinitis						
No	91 (46.43)	103 (52.55)	0.224	78 (39.80)	116 (59.18)	0.165
Yes	2 (1.02)	0 (0)		2 (1.02)	0 (0)	
Nasal irrigation						
No	72 (36.73)	39 (19.90)	<**.001**	61 (31.12)	55 (28.06)	<**.001**
Yes	21 (10.71)	64 (32.65)		19 (8.16)	66 (33.67)	

A p < 0.05 is highlighted in bold.

Abbreviations: RT, radiotherapy.

a According to the Union for International Cancer Control/American Joint Committee on Cancer criteria (8th version).

**Table 2:** Dosimetric parameters during VMAT of maxillary sinus.

Variable	Median (range)
Volume of maxillary sinus (cm^3^)	24.2 (2.1, 66.9)
% of maxillary sinus volume receiving 20Gy (%)	100.0 (91.3, 100.0)
% of maxillary sinus volume receiving 30Gy (%)	100.0 (51.0, 100.0)
% of maxillary sinus volume receiving 40Gy (%)	83.0 (3.0, 100.0)
% of maxillary sinus volume receiving 50Gy (%)	51.0 (8.5, 100.0)
% of maxillary sinus volume receiving 60Gy (%)	15.5 (10.0, 99.1)
% of maxillary sinus volume receiving 70Gy (%)	0.0 (0.0, 27.6)
Mean dose received by maxillary sinus (Gy)	49.9 (31.1, 66.9)
Minimum dose received by maxillary sinus (Gy)	29.7 (10.2, 59.3)
Maximum dose received by maxillary sinus (Gy)	68.7 (44.5, 75.1)
Median dose received by maxillary sinus (Gy)	49.9 (31.0, 67.0)

### 3.2 Univariate Cox regression analysis for post-RT rhinosinusitis

The association of selected clinical characteristics with post-RT rhinosinusitis are shown in Table 3. Univariate Cox regression analysis showed that nasal irrigation was negatively associated with post-RT rhinosinusitis (HR = 0.31[0.19, 0.51], p < 0.001). Age, sex, N stage, chemotherapy regime, hypertension, diabetes mellitus, alcohol use, smoking status, and treatment regime were not found to be independent risk factors for radiation-induced rhinosinusitis.

**Table 3:** Univariate Cox regression model for radiation-induced rhinosinusitis.

Variable	Univariate analysis Number (%)	
	HR (95 % CI)	p
Age (y)	0.99 (0.98, 1.01)	0.451
Sex		
Male	1	
Female	1.01 (0.65, 1.58)	0.956
Tumor stage^a^		
T1-2	1	
T3-4	1.41 (0.91, 2.19)	0.126
Node stage^a^		
N0-1	1	
N2-3	1.05 (0.69, 1.58)	0.828
Overall stage^a^		
I-Ⅱ	1	
Ⅲ-Ⅳ	1.43 (0.81, 2.52)	0.221
Treatment regime		
RT alone	1	
Chemoradiotherapy	1.45 (0.73, 2.89)	0.286
Alcohol consumption		
No	1	
Yes	1.12 (0.60, 2.11)	0.719
Smoker		
No	1	
Yes	0.88 (0.57, 1.37)	0.570
Family history of cancer		
No	1	
Yes	0.98 (0.48, 1.99)	0.960
Hypertension		
No	1	
Yes	1.32 (0.48, 3.64)	0.591
Diabetes mellitus		
No	1	
Yes	0.78 (0.11, 5.63)	0.806
Nasal irrigation		
No	1	
Yes	0.31 (0.19, 0.51)	<**.001**

A p < 0.05 is highlighted in bold.

Abbreviations: RT, radiotherapy.

a According to the Union for International Cancer Control/American Joint Committee on Cancer criteria (8th version).

### 3.3 Univariate and multivariate cox regression analyses for post-RT maxillary sinusitis

Nasal irrigation was found to be negatively associated with maxillary sinusitis on univariate analysis (HR = 0.24 [0.13, 0.44], p < 0.001). In contrast, V60 and V70 were significantly associated with higher odds of development of maxillary sinusitis. Other clinical factors such as age, sex, N stage, chemotherapy regime, hypertension, diabetes mellitus, alcohol use, smoking status, and treatment regime and dosimetric parameters such as maxillary sinus volume, Dmean (maxillary sinus), Dmax (maxillary sinus), V20, V30, V40, and V50 did not reach statistical significance. In the multivariate analysis, the effects of nasal irrigation (HR = 0.23 [0.12, 0.44], p < 0.001) and V70 (HR = 1.12 [1.01, 1.16], p = 0.027) for maxillary sinusitis remained statistically significant (Table 4). The cut-off point for the V70 level for the incidence of maxillary sinusitis was 1.16 % with the standardized log-rank statistics using exact Gauss (Fig. 1C). We further classified the patients into the following groups according to the cut-off values and the incidence of radiation-induced rhinosinusitis in the group with V70 ≤ 1.16 % and V70 > 1.16 % was 35.62 % and 63.88 % respectively. Patients with V70 > 1.16 % had significantly higher cumulative incidence rates of maxillary sinusitis than patients with V70 ≤ 1.16 % (p < 0.01) (Fig. 1D).

**Table 4:** Univariate and multivariate cox regression model of radiation-induced maxillary sinusitis.^a^.

Variable	Univariate analysis		Multivariate analysis	
	HR (95 % CI)	p	HR (95 % CI)	p
Age	1.28 (0.69, 2.40)	0.433		
Sex	0.99 (0.97, 1.02)	0.491		
Tumor stage*	1.65 (0.90, 3.02)	0.104		
Node stage*	1.62 (0.52, 5.03)	0.408		
Overall stage*	0.17 (0.01, 2.05)	0.172		
RT alone	1.51 (0.62, 3.67)	0.036		
Alcohol	0.04 (0.57, 1.90)	0.092		
Smoker	1.01 (0.55, 1.84)	0.985		
Hypertension	1.48 (0.41, 5.29)	0.646		
Diabetes mellitus	1.46 (0.20, 10.60)	0.707		
Nasal irrigation	0.24 (0.13, 0.44)	<**.001**	0.23 (0.12, 0.44)	<**.001**
DVH parameters				
Total volume	1.04 (0.99, 1.09)	0.163		
V20Gy	0.56 (1.67, 1.91)	0.356		
V30Gy	0.99 (0.94, 1.05)	0.682		
V40Gy	1.00 (0.97, 1.02)	0.746		
V50Gy	1.00 (0.99, 1.02)	0.805		
V60Gy	1.04 (1.01, 1.07)	**0.008**	1.03 (0.99, 1.07)	0.094
V70Gy	1.18 (1.03, 1.35)	**0.017**	1.12 (1.01, 1.16)	**0.027**
Dmean	0.98 (0.95, 1.02)	0.359		
Dmin	0.99 (0.95, 1.03)	0.506		
Dmax	1.00 (1.00, 1.01)	0.080		
Dmed	1.01 (0.90, 1.02)	0.937		

A p < 0.05 is highlighted in bold.

Abbreviations: RT, radiotherapy.

a According to the Union for International Cancer Control/American Joint Committee on Cancer criteria (8th version).

### 3.4 Univariate and multivariate analysis of maxillary sinus dosimetric factors for post-RT ethmoid sinusitis, sphenoid sinusitis, and frontal sinusitis

Our analysis extended to evaluate the impact of maxillary sinus dosimetric factors on the development of post-RT ethmoid, sphenoid, and frontal sinusitis. Dose-volume histogram parameters of the maxillary sinus, specifically Dmean (maxillary sinus), Dmax (maxillary sinus), and Dmed (maxillary sinus), along with V50, V60, and V70, were predictive of ethmoid sinusitis in univariate analysis. V70 maintained statistical significance in multivariate analysis (OR = 1.25 [1.08, 1.44], p = 0.002) (Table S1). For sphenoid sinusitis, Dmax (maxillary sinus) emerged as a significant multivariate factor (OR = 1.00 [0.99, 1.01], p = 0.005) (Table S2). Although various parameters correlated with frontal sinusitis on univariate analysis, multivariate analysis did not reveal significant associations (Table S3).

**Fig. 3:**
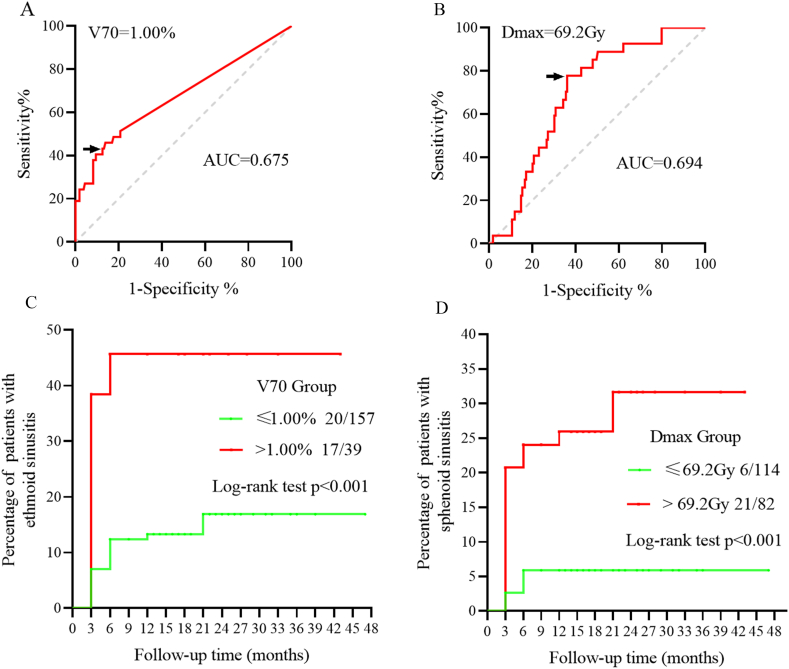
(A) ROC analysis of V70. (B) ROC analysis of Dmax. (C) The cumulative incidence of ethmoid sinusitis was classified by the level of (A) V70. (D) The cumulative incidence of ethmoid sinusitis was classified by the level of (B) Dmax.

The ROC curve analysis established a threshold of 1.00 % for V70 in predicting ethmoid sinusitis (p = 0.001) (Fig. 3A), and a Dmax (maxillary sinus) threshold of 69.2Gy for sphenoid sinusitis (p = 0.001) (Fig. 3B). Kaplan-Meier survival curves reinforced these findings, showing a marked increase in the cumulative incidence of ethmoid sinusitis when V70 exceeded 1.00 % (43.6 % vs. 12.7 %, p < 0.001) (Fig. 3C), and a similar trend for sphenoid sinusitis with a Dmax over 69.2Gy (25.6 % vs. 5.3 %, p < 0.001) (Fig. 3D).

## 4 Discussion

Maintaining normal sinonasal physiology requires functional mucus production, normal mucociliary function, unimpaired flow of secretions through the sinus ostia and outflow pathways and ventilation of the sinuses. Rhinosinusitis is an inflammation and/or infection of the paranasal sinuses due to disruption of the factors above. Radiation has been shown to lead to damage sinonasal mucosa, leading to altered secretions, impaired mucociliary function and oedema leading to obstruction of sinus outflow tracts [8,9]. Identifying potentially modifiable factors that predispose to post-RT rhinosinusitis and interventions that can minimize rhinosinusitis are the goals of this study.

Our study identified the clinical and dosimetric risk factors, as well as the dose-volume threshold values associated with radiation-induced rhinosinusitis in NPC patients treated with VMAT. While it had been demonstrated that different RT modalities and parameters affect the incidence of sinonasal complications [5,7], VMAT achieved better target dose distribution and similar sparing of critical structures than various RT modalities [13]. This is the possibly the first study demonstrating an association between post-radiation rhinosinusitis and VMAT dosimetric parameters in NPC patients.

In comparing various RT modalities, while the incidence of rhinosinusitis following VMAT (47.4 %) had been lower compared with conventional RT and IMRT [5,7]. Therefore, it indicates that VMAT may show a significant dose advantage in protecting the normal sinus tissue. In our study, both univariate and multivariate analyses identified nasal irrigation as a protective factor against radiation-induced rhinosinusitis, which corroborated with the findings of previous studies [7,18]. From a physiologic point of view, nasal irrigation may help restore the normal physiology of nasal mucosa and remove debris and crusts [19]. This has been our clinical observation and our study results reinforce the benefits of encouraging patients to perform routine nasal irrigation after radiotherapy.

Intriguingly, our data did not substantiate a relationship between NPC stage and rhinosinusitis incidence, potentially due to the small number of early-stage cases and the protective effect of VMAT on sinus tissue. Wu et al. demonstrated that chemotherapy was not significantly different between patients with and without rhinosinusitis [7]. Our results support the former conclusion. Contradictory to previous findings [11], smoking was not identified as a risk factor in our cohort, suggesting that VMAT may offset some traditional risk exposures. We further observed a rapid increase in post-RT rhinosinusitis incidence within the first 3–6 months after VMAT, which subsequently trended towards a plateau after 1 year. These findings were also consistent with those reported in the literature [7,20,21]. We advocate adopting nasal irrigation in patient who underwent VMAT within 3–6 months to reduce the incidence of rhinosinusitis.

Like previous studies [7,21], our study found maxillary sinusitis was the most frequently diagnosed form of post-RT rhinosinusitis. We postulate that this could be due to the dependent anatomy of the maxillary sinus with its ostium located superiorly, rendering normal ciliary action for mucus removal against gravity a necessity, and RT has been shown to damage or impair ciliary function [8]. Also, the osteomeatal complex (OMC) through which the maxillary sinus drains is a narrow area that can be obstructed easily due to oedema and crusting. The data presented here is a large data set that evaluates the relationship between various treatment parameters and development of maxillary sinusitis after VMAT for NPC. The most obvious finding emerging from this analysis is that V70 is significantly correlated with maxillary sinusitis, retaining its statistical significance in the multivariate analysis. Our data suggests that the percentage of maxillary sinus receiving 70Gy should be limited where possible such that V70 ≤ 1.16 % in order to reduce the incidence of maxillary sinusitis. This finding may aid in guiding individualized treatment planning and reducing the incidence of radiation-induced rhinosinusitis.

Extending the scope of analysis, we observed that maxillary sinus dosimetric factors influenced the development of ethmoid and sphenoid sinusitis. A V70 ≤ 1.00 % appeared to be a protective threshold for ethmoid sinusitis, while a Dmax(maxillary sinus) > 69.2Gy was identified as a risk factor for sphenoid sinusitis. However, similar dosimetric influences on frontal sinusitis were not established, indicating that the geometric factors may play the major role in the different dosiometric factors associations. The ethmoid sinus is in close proximity to the maxillary sinus, thus sharing the same V70 as the sensitive dosimetric factor as maxillary sinusitis. Meanwhile, the sphenoid sinus is further away, and a stricter Vmax is needed to predict sphenoid sinusitis. Similarly, the frontal sinus is much further away from the maxillary sinus. Therefore, no dosimetric factor from the maxillary sinus is predictive to frontal sinusitis.

Our study had several limitations. First, it was a retrospective study based solely on clinical observations from patients of a single tertiary treatment center. Subsequent prospective multi-center studies are needed to verify our conclusions. In addition, while the recommended dosimetric restrictions for the maxillary sinus is achieved during treatment planning, this does not ensure that sinusitis does not occur at other sites. Thus, a more comprehensive dose reduction approach is necessary to fully address this issue. Lastly, the effects of VMAT on the paranasal sinuses were assessed based on the presence or absence of rhinosinusitis and the severity of rhinosinusitis was not assessed.

In conclusion, nasal irrigation may be protective against radiation-induced rhinosinusitis following VMAT for NPC. VMAT appears to confer an advantage over 3D conformal RT and IMRT in reducing rhinosinusitis incidence. Dose-volume constraints such as V70 and Dmax to the maxillary sinus have emerged as valuable predictors of this complication. These insights provide a foundation for clinical guidelines aimed at minimizing the development of RT-related rhinosinusitis, highlighting the importance of timely interventions within the first six months following VMAT.

## Funding information

The 10.13039/501100001809National Natural Science Foundation of China, Grant/Award Number: 81974141; the 10.13039/501100003453Guangdong Natural Science Foundation, Grant/Award Number: 2022A1515010506.

## Data availability statement

The data for this article is accessible and has been deposited in Mendeley Data, V1, https://doi.org/10.17632/6v43kt633p.1.

## Ethics statement

This study was reviewed and approved by IEC for Clinical Research and Animal Trials of Sun Yat-sen University, with the approval number: 2022265. Informed consent was not required for this study because data were acquired anonymously with no identification information.

## CRediT authorship contribution statement

**Xiaomin Bao:** Writing – review & editing, Writing – original draft, Software, Methodology, Investigation, Formal analysis, Data curation. **Yan Wang:** Resources, Investigation, Data curation. **Bin Li:** Software, Formal analysis, Data curation. **Liang Peng:** Software, Formal analysis, Data curation. **Bin Ouyang:** Investigation, Formal analysis, Data curation. **Chew Lip Ng:** Writing – review & editing, Writing – original draft. **Yongshi Zhuo:** Data curation. **Qiumin Wang:** Data curation. **Chunwei Li:** Writing – review & editing, Writing – original draft, Visualization, Validation, Supervision, Project administration, Conceptualization. **Jian Li:** Writing – review & editing, Writing – original draft, Visualization, Validation, Supervision, Project administration, Funding acquisition, Conceptualization.

## Declaration of competing interest

The authors declare that they have no known competing financial interests or personal relationships that could have appeared to influence the work reported in this paper.
