# Supplementary material for: Clinical and dosimetric predictors of radiation-induced rhinosinusitis following VMAT for nasopharyngeal carcinoma: A retrospective study
Source: Heliyon. 2023 Dec 12;10(1):e23554. doi: 10.1016/j.heliyon.2023.e23554 (PMC10770446; doi:10.1016/j.heliyon.2023.e23554)
Supplement: Multimedia component 1 [file mmc1.docx]

**TABLE S1** Univariate and multivariate analysis for maxillary sinus dosimetric factors to the development of ethmoid sinusitis

| **Variable** | **Univariate analysis** | |  | **Multivariate analysis** | |
| --- | --- | --- | --- | --- | --- |
|  | **OR (95% CI)** | **p** |  | **OR (95% CI)** | **p** |
| V20Gy | 9.68 (-) | 1.000 |  |  |  |
| V30Gy | 1.08 (0.94, 1.24) | 0.262 |  |  |  |
| V40Gy | 1.03 (1.00, 1.05) | 0.055 |  |  |  |
| V50Gy | 1.02 (1.01, 1.04) | **0.003** |  | 1.00 (0.98, 1.02) | 0.765 |
| V60Gy | 1.04 (1.01, 1.06) | **0.003** |  | 0.99 (0.95, 1.03) | 0.673 |
| V70Gy | 1.28 (1.13, 1.45) | **<.001** |  | 1.25 (1.08, 1.44) | **0.002** |
| Dmean | 1.00 (1.00, 1.00) | **0.002** |  |  |  |
| Dmin | 1.00 (1.00, 1.00) | 0.208 |  |  |  |
| Dmax | 1.00 (1.00, 1.00) | **0.006** |  | 1.00 (1.00, 1.00) | 0.114 |
| Dmed | 1.00 (1.00, 1.00) | **0.004** |  |  |  |

A p < 0.05 is highlighted in bold.

**TABLE S2** Univariate and multivariate analysis for maxillary sinus dosimetric factors to the development of sphenoid sinusitis

| **Variable** | **Univariate analysis** | |  | **Multivariate analysis** | |
| --- | --- | --- | --- | --- | --- |
|  | **OR (95% CI)** | **p** |  | **OR (95% CI)** | **p** |
| V20Gy | 9.28 (-) | 1.000 |  |  |  |
| V30Gy | 1.34 (0.95, 1.89) | 0.099 |  |  |  |
| V40Gy | 1.04 (1.01, 1.08) | **0.019** |  | 1.01 (0.96, 1.06) | 0.643 |
| V50Gy | 1.02 (1.01, 1.04) | **0.006** |  | 1.01 (0.98, 1.04) | 0.508 |
| V60Gy | 1.04 (1.01, 1.06) | **0.004** |  | 1.00 (0.96, 1.04) | 0.889 |
| V70Gy | 1.12 (1.03, 1.23) | **0.011** |  | 1.04 (0.91, 1.19) | 0.553 |
| Dmean | 1.00 (1.00, 1.00) | **0.012** |  | 1.00 (0.99, 1.00) | 0.983 |
| Dmin | 1.00 (1.00, 1.00) | 0.115 |  |  |  |
| Dmax | 1.00 (1.00, 1.00) | **0.003** |  | 1.00 (0.99, 1.00) | **0.005** |
| Dmed | 1.00 (1.00, 1.00) | **0.034** |  | 1.00 (0.99, 1.00) | 0.766 |

A p < 0.05 is highlighted in bold.

**TABLE S3** Univariate and multivariate analysis for maxillary sinus dosimetric factors to the development of frontal sinusitis

| **Variable** | **Univariate analysis** | |  | **Multivariate analysis** | |
| --- | --- | --- | --- | --- | --- |
|  | **HR (95% CI)** | **p** |  | **HR (95% CI)** | **p** |
| V20Gy | 7.84 (-) | 1.000 |  |  |  |
| V30Gy | 1.79 (0.49, 6.43) | 0.374 |  |  |  |
| V40Gy | 1.11 (1.00, 1.23) | **0.049** |  | 1.02 (0.92, 1.13) | 0.713 |
| V50Gy | 1.04 (1.01, 1.08) | **0.017** |  | 1.04 (0.97, 1.11) | 0.269 |
| V60Gy | 1.04 (1.01, 1.07) | **0.023** |  | 0.95 (0.89, 1.02) | 0.189 |
| V70Gy | 1.17 (1.05, 1.30) | **0.004** |  | 1.06 (0.86, 1.31) | 0.597 |
| Dmean | 1.00 (1.00, 1.00) | 0.143 |  |  |  |
| Dmin | 1.00 (1.00, 1.00) | **0.002** |  | 1.00 (1.00, 1.00) | 0.107 |
| Dmax | 1.00 (1.00, 1.00) | 0.061 |  |  |  |
| Dmed | 1.00 (1.00, 1.00) | 0.070 |  |  |  |

A p< 0.05 is highlighted in bold.
